# ER Stress Activates the TOR Pathway through Atf6

**DOI:** 10.5334/1750-2187-13-1

**Published:** 2018-04-23

**Authors:** Dylan Allen, Jin Seo

**Affiliations:** 1 Department of Biology, School of Arts and Sciences, Rogers State University, Claremore, OK, US

**Keywords:** ER stress, TOR pathway, Atf6

## Abstract

Cellular signaling pathways are often interconnected. They accurately and efficiently regulate essential cell functions such as protein synthesis, cell growth, and survival. The target of rapamycin (TOR) signaling pathway and the endoplasmic reticulum (ER) stress response pathway regulate similar cellular processes. However, the crosstalk between them has not been appreciated until recently and the detailed mechanisms remain unclear. Here, we show that ER stress-inducing drugs activate the TOR signaling pathway in S2R+ *Drosophila* cells. Activating transcription factor 6 (Atf6), a major stress-responsive ER transmembrane protein, is responsible for ER stress-induced TOR activation. Supporting the finding, we further show that knocking down of both site-1/2 proteases (S1P/S2P), Atf6 processing enzymes, are necessary to connect the two pathways.

## Introduction

Intracellular signaling pathways are arranged as complex interrelated networks and determine virtually all aspects of essential cell functions including gene expression, mRNA translation, and proliferation; thus, dysregulation of signaling networks will severely damage cell viability.

The endoplasmic reticulum (ER) is a membranous network in the cytoplasm of the cell. The extensive ER luminal space is the site of protein modification, lipid synthesis and Ca^2+^ storage. Overload of protein synthesis or Ca^2+^ imbalance in the ER lumen could cause stress in this organelle and lead to cellular dysfunction [1,2,3]. To cope with the stress, cells have developed a machinery called the ER stress response pathway or the unfolded protein response (UPR) pathway. Upon ER stress, perturbation of the ER is detected by three ER transmembrane protein sensors: inositol requiring enzyme 1 (IRE1), pancreatic ER kinase (PERK), and activating transcription factor 6 (Atf6). Each sensor independently activates its unique and overlapping targets to recover ER homeostasis. Upon ER stress, IRE1 is activated by its oligomerization to become an active endonuclease [4]. The active IRE1 endonuclease digests multiple mRNAs and reduces new protein synthesis [5] in the process known as regulated IRE1-dependent mRNA decay (RIDD). Simultaneously, activated IRE1 removes an unconventional intron within X-box binding protein-1 (XBP1) mRNA in the cytoplasm [6,7]. This splicing produces a functional XBP1, thus enhancing gene expression of ER-associated degradation (ERAD) proteins and ER chaperones. The second sensor, PERK is activated during ER stress. Once activated, PERK phosphorylates eukaryotic initiation factor 2a (eIF2a), thereby inhibiting general protein synthesis [8]. Phosphorylated-eIF2a binds to and sequesters eIF2B [9], an essential translation accessary component, which converts inactive eIF2-GDP to active eIF2-GTP. Thus, eIF2-GTP is depleted, thereby inhibiting formation of eIF2-tRNAi^Met^-mRNA tertiary complex, which results in an attenuation of general translation [10]. Accordingly, both IRE1 and PERK relieve ER stress by decreasing the load of protein synthesis entering the ER. The third ER sensor, Atf6, is delivered to the Golgi apparatus upon accumulation of unfolded/misfolded proteins; there, Atf6 is digested and activated by two S1P/S2P Golgi proteases, thereby allowing the cytoplasmic bZIP domain to translocate into the nucleus and act as a transcription factor [11]. Activated Atf6 transcription domain upregulates molecular chaperones such as GRP78, thereby increasing ER protein-folding capacity [12]. Multiple knock out mouse models of the UPR pathway have demonstrated that the UPR pathway controls obesity, energy metabolism, and pancreatic beta cell survival [13,14,15,16,17].

The target of rapamycin (TOR) pathway also regulates multiple cellular processes involved in mRNA translation, proliferation, growth, and metabolism [18,19,20]. TOR exists as multi-protein complexes called TOR complex 1 (TORC1) or TORC2. TORC1 contains regulatory-associated protein of TOR (Raptor), DEP-domain-containing TOR-interacting protein (Deptor), mammalian lethal with Sec13 protein 8 (mLST8), proline-rich AKT substrate 40 kD (PRAS40), and TOR kinase. TORC2 was originally identified as a regulator of actin cytoskeleton organizer [21]. However, comprehensive cellular function and activation mechanism of TORC2 are poorly understood. In addition, only TORC1 but not TORC2 is sensitive to rapamycin, a small molecular inhibitor. In this paper, we refer to TORC1 as TOR unless stated otherwise. TORC1 activity is regulated by several upstream modifiers such as ras homolog enriched in brain (Rheb) and the complex of tuberous sclerosis complex 1 (TSC1) and TSC2. Rheb, a small GTPase, activates TORC1 while TSC1/2 inhibits TORC1. TORC1 regulates multiple downstream targets including ribosomal protein S6 kinase (S6K) and eukaryotic initiation factor 4E binding protein 1 (4E-BP1) [20]. The TOR pathway promotes many anabolic processes such as protein synthesis, lipid metabolism, and organelle biogenesis, while restricting catabolic processes such as the degradation of protein complexes and organelles. The TOR pathway and ER stress pathway have shown similar effects on multiple cellular processes including mRNA translation and lipid metabolism [22].

Individually, the UPR and the TOR pathways have been extensively studied; however, to date a detailed mechanism of crosstalk between the two pathways remains unclear. Recently, however, the TOR pathway has been shown to play a crucial role controlling ER stress-induced apoptosis, [23,24] and activating transcription factor 4 (Atf4), an essential regulator of ER stress, has been shown to regulate TOR signaling [17]. Further, certain cancer cells can be effectively treated by controlling the two signaling pathways [25,26] supporting the notion of crosstalk between the UPR and TOR pathways. Here, using S2R+ *Drosophila* cells, we show that ER stress-inducing reagents activate the TOR pathway. Further, through gain and loss of function experiments, we find that Atf6 is responsible for activating TOR signaling. Supporting this finding, we report that S1P/S2P proteases, which activate Atf6, are necessary to regulate the TOR in response to ER stress.

## Materials and Methods

### Tissue Culture

The *Drosophila melanogaster* S2R+ cells [27] were cultured in Schneider’s *Drosophila* medium supplemented with 10% FBS and 1x penicillin-streptomycin-glutamine. The cells were maintained in a 25°C incubator with air. Phosphate buffered saline (PBS) was used as nutrient free media (NF-M).

### Double Strand RNA (dsRNA) Synthesis and Transfection

Both forward and reverse oligonucleotides of target genes were designed using SnapDragon-dsRNA Design tool (http://www.flyrnai.org). Then, the T7 polymerase-binding sequence (taatacgactcactataggg) was flanked to the 5’ end of each primer. Using the primer sets, the target genes were amplified by standard PCR procedure; target dsRNAs were generated using the PCR product and *in vitro* T7 transcription kit (Megascript). Firefly luciferase gene (Luc) was used to generate control dsRNA. S2R+ cells were transfected with the dsRNA (1–2 mg) using FuGENE 6 (Promega) and were incubated for 2 days. Then, the transfected cells were collected for further analysis. Both primer sequences of target genes are shown in the following Table 1.

**Table 1:** Primer sequences to generate dsRNAs.

Primer Name	Sequence

Luc-F	taatacgactcactatagggAGATGGAACCGCTGGAGAGC
Luc-R	taatacgactcactatagggGACTCTGGCACAAAATCG
PERK-F	taatacgactcactatagggTGGCACAAGGAGGGGAAC
PERK-R	taatacgactcactatagggGCACCACTGGACCTAGTAAA
IRE1-F	taatacgactcactatagggCAAAAGCAGAGCGAGAATG
IRE1-R	taatacgactcactatagggTTAATGTCGCGATGCACAA
Atf6-I-F	taatacgactcactatagggAACATTACGGGATCAGATATTTC
Atf6-I-R	taatacgactcactatagggAAGGGTGGAGGTTCATTATATG
Atf6-II-F	taatacgactcactatagggCTTCAGCGTGTTCTGGTGAA
Atf6-II-R	taatacgactcactatagggGTTGGCAAAAGCCCTTTGTA
S1P-F	taatacgactcactatagggAAAGGTGTTGGAACTGTCGG
S1P-R	taatacgactcactatagggGGTATGCCGGAATAAGGGTT
S2P-F	taatacgactcactatagggGACAGAATTTTCGCGGAGAG
S2P-R	taatacgactcactatagggTGAGGTGCTACAATTCGCTG
TSC1-F	taatacgactcactatagggCAGCCTGCCAGAAATTAACT
TSC1-R	taatacgactcactatagggCCAGTCCTTCCACCGTCT

### Western Blotting and Antibodies

Western blotting was performed according to standard procedure. In brief, protein extracts of S2R+ cells were prepared using PhosphoSafe Extraction Reagent (EMD Millipore) to inhibit phosphatases, mixed with 2X Laemmli sample buffer (Bio-Rad), boiled, separated on SDS-polyacrylamide gel, and transferred onto nitrocellulose membrane. The nitrocellulose membrane was blocked with 5% milk in tris-buffered saline (TBS), or blocking buffer (LI-COR) and then, incubated with the primary antibody. Subsequently, the membrane was washed with TBS containing 0.1% tween 20 (TBST) and incubated with secondary antibody conjugated with HRP or fluorescent dye. We used the Odyssey Fc imaging system (LI-COR) to detect both chemiluminescent and fluorescent signals from secondary antibody. All antibodies were diluted with TBST containing 1% milk or blocking buffer with the indicated dilution: anti-phosphor(Thr398)-S6K (Cell Signaling Technology #9203, 1:1,000), anti-myc (Cell Signaling Tech #2276,1:1,000), anti-a-tubulin (Cell Signaling Technology #2144,1:2,500), HRP-anti-mouse (LI-COR #926-80010, 1:5,000), HRP-anti-rabbit (LI-COR #926-80011,1:5,000), IRDye 680RD-anti-mouse (LI-COR #926-68170, 1:10,000), and IRDye 800CW-anti-rabbit (LI-COR #925-32211,1:10,000).

### RNA Extractions and Reverse-Transcriptase PCR

Total RNA was extracted using the RNeasy purification kit (Qiagen). To generate cDNA, 1 mg of total RNA was reverse-transcribed with Moloney Murine Leukemia Virus Reverse Transcriptase (M-MLV RT, Thermo Fisher Scientific) and random hexamers (Thermo Fisher Scientific). The presence of transfected dsRNA and knocking down of endogenous mRNA were measured by PCR using different sets of primers. Then, the products were separated by agarose gel electrophoresis. Total and spliced forms of XBP1 were detected using the following primers (Table 2) as described previously [28].

**Table 2:** Primer sequences to quantitate total and spliced XBP1.

Primer Name	Sequence

T-XBP1-F	ATACGCATCCTCGTCGAACATGGATGAC
T-XBP1-R	TCATCTAGAAAAACTCAGATCAAACTGG
S-XBP1-F	CCGAATTCAAGCAGCAACAGCA
S-XBP1-R	TAGTCTAGACAGAGGGCCACAATTTCCAG

### Chemicals

ER stress-inducing drugs and other chemicals were dissolved in indicated solvents and used as the final concentrations in the parentheses. Dithiothreitol (Sigma-Aldrich, 1 μg/ml) was dissolved with distilled water; thapsigargin (Sigma-Aldrich, 0.2 μM) and tunicamycin (Sigma-Aldrich, 0.5 μg/ml) were dissolved with DMSO. Bovine insulin (Sigma-Aldrich, 10 μg/ml) and rapamycin (LC Lab, 27 μM) were diluted with the growth media to obtain the final concentrations in parentheses.

## Results

### Both ER stress and TOR pathways are functional in S2R+ cells

**Figure 1:**
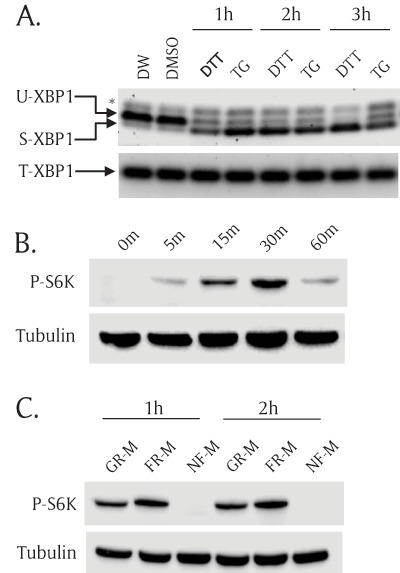
**Both ER stress and TOR pathways are functional in S2R+ *Drosophila* cells. (A)** ER stress inducers activated XBP1 mRNA splicing in S2R+ *Drosophila* cells. S2R+ cells were incubated with ER stress inducers for 1 hour, 2 hours, and 3 hours; then, total RNA was extracted and used to generate cDNA. Unspliced XBP1(U-XBP1) and spliced XBP1 (S-XBP1) were amplified using specific primer set across the XBP1 splicing region. Total XBP1 (T-XBP1) was used as a loading control. Asterisk (*) indicates a non-specific band. 1 mM dithiothreitol (DTT) and 0.2 μM thapsigargin (TG). Distilled water (DW) is the vehicle for DTT and Dimethylsulfoxide (DMSO) is the vehicle for TG. **(B)** Insulin increased phosphorylation of S6K. S2R+ cells were incubated with insulin (10 mg/ml) for multiple incubation times: 0 minutes, 5 minutes, 15 minutes, 30 minutes, and 60 minutes. The cell lysates were subjected to SDS-PAGE and transferred to nitrocellulose paper followed by Western blot analysis. Phospho(Thr398)-specific S6K antibody (P-S6K) detects only phosphorylated form of S6K. Tubulin serves as a loading control. **(C)** Nutrient-free (NF-M) media completely abolished S6K phosphorylation. S2R+ cells were cultured in growth media (GR-M) for 1 day. Then the cells were incubated with fresh media (FR-M) or nutrient free-media (NF-M) for 1 hour and 2 hours. Phospho(Thr398)-S6K and tubulin were detected by Western blot analysis.

Using S2R+ *Drosophila* cells, we investigated possible crosstalk between two functionally-related signaling pathways: the ER stress response pathway and the TOR pathway. We first tested whether ER stress-inducing drugs could elicit splicing of XBP1 mRNA, a hallmark of ER stress [6]. We treated S2R+ cells with ER stress inducing drugs, extracted total RNA from the cells, and generated their cDNA to test XBP1 splicing. We used two XBP1 primer sets to quantitate both total and spliced XBP1. The first primer set amplifies a region across the 23 bp-splicing site to distinguish between the spliced form of XBP1 (220 bp) and the unspliced form (240 bp); the other set amplifies outside of the splicing region, thus amplifying the same size of XBP1 regardless of XBP1 splicing [28]. The spliced form of XBP1 was significantly increased in ER stress-induced samples while the total amount of XBP1 remained the same (Figure 1A). Furthermore, dithiothreitol (DTT) increased the spliced form of XBP1 in a time-dependent manner. Unspliced XBP1 mRNA was completely abolished while the amount of the spliced form peaked three hours after DTT incubation. However, thapsigargin (TG) did not further increase the spliced-form with longer drug treatment (Figure 1A).

We then tested whether the TOR pathway was also functional in S2R+ cells. We treated S2R+ cells with insulin or nutrient-free media (NF-M) and measured TOR signaling activity by assessing phosphorylation (Thr398) of S6K (P-S6K), a well-characterized TOR target. Insulin treatment increased P-S6K which reached a peak 30 minutes after insulin incubation (Figure 1B). At one hour time point, reduction of P-S6K is likely due to a negative feedback inhibition of insulin signaling pathway by prolonged activation of S6K [29,30,31]. By contrast, a nutrient-free media (NF-M) completely abolished phosphorylation of S6K (Figure 1C). These results demonstrate that both ER stress and the TOR pathways are functional in S2R+ cells.

### ER stress inducers activate the TOR pathway

**Figure 2:**
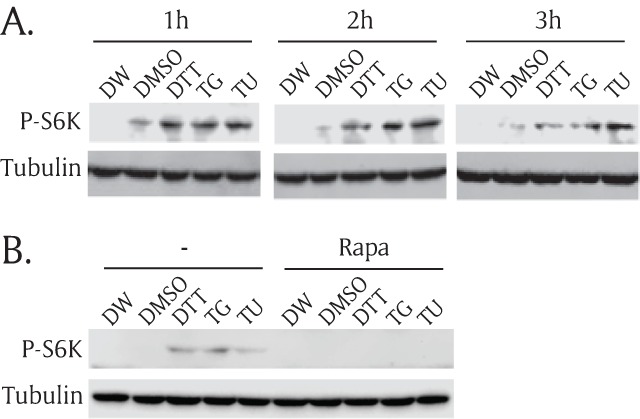
**ER stress inducers activate the TOR pathway. (A)** S2R+ cells were incubated in nutrient-free media for one hour, and then treated with three ER stress inducers in serum-free growth media for 1 hour, 2 hours, and 3 hours along with two vehicle controls. Then, cell lysates were prepared, subjected to SDS-PAGE, transferred to nitrocellulose paper, and then incubated with phospho(Thr398)-specific S6K (P-S6K) antibody or tubulin antibody. Distilled water (DW) was used as a vehicle for 1 mM dithiothreitol (DTT). The same amount of dimethylsulfoxide (DMSO) was used as a vehicle control for 0.2 μM thapsigargin (TG) and 0.5 mg/ml tunicamhycin (TU). **(B)** S2R+ cells were incubated in nutrient-free media for one hour and then, treated for another one hour with three ER stress inducers with or without 27 μM rapamycin (Rapa). Then, the cell lysates were subjected to Western blot.

To test whether ER stress can regulate the TOR signaling pathway, we incubated S2R+ cells with ER stress inducers and evaluated TOR signaling activity. We used three different ER-stress inducers: DTT, TG, and tunicamycin (TU), for multiple incubation time points. The cells were incubated with a nutrient-free media, treated with ER stress inducers, collected, lysed, and measured for TOR activity by Western blots. Deionized water (DW) served as a vehicle control for DTT while dimethyl sulfoxide (DMSO) served as a vehicle control for TG and TU. Despite the use of different mechanisms of the three ER stress inducers, all three stress inducers increased the phosphorylation of S6K as early as after one hour of incubation and the levels of P-S6K were maintained for at least three hours (Figure 2A). DMSO vehicle control periodically increased phosphorylation of S6K comparing to the DW control. To further test whether the TOR kinase is responsible for ER stress-induced phosphorylation of S6K, we then incubated ER stress inducers together with rapamycin, a potent TOR kinase inhibitor. Rapamycin completely blocked ER stress-induced phosphorylation of S6K (Figure 2B). These data demonstrate that acute ER stress activates the phosphorylation of S6K via the TOR kinase. DTT showed the strongest effect on the ER stress-mediated TOR activation and thus, we used DTT for further experiments to induce ER stress.

### Knock down of Atf6 inhibits TOR signaling

**Figure 3:**
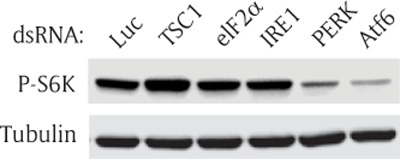
**Knock down of Atf6 inhibits TOR signaling.** S2R+ cells were transfected with luciferase (Luc), TSC1, eIF2a, IRE1, PERK, or Atf6 double-stranded (ds) RNA. The transfected cells were cultured for 2 days, and incubated with serum-free media for 1 hour before cells were collected. Then, Western blot analysis was performed with phospho(Thr398)-specific S6K antibody or tubulin antibody. Firefly luciferase (Luc), Tuberous sclerosis complex 1 (TSC1), Eukaryotic initiation factor 2a (eIF2a), Inositol requiring enzyme 1 (IRE1), Pancreatic ER kinase (PERK), and Activating transcription factor 6 (Atf6).

There are three main ER stress sensors in the ER membrane: IRE1, PERK, and Atf6. The sensors detect ER stress and transduce the perturbation signals across the ER to maintain ER homeostasis by inhibiting global mRNA translation, degrading unfolded/misfolded proteins, and increasing expression of molecular chaperones [1,2,3]. We attempted to determine which ER sensor is necessary to elicit activation of the TOR signaling in normal growth conditions without acute ER stress. To this end, we individually knocked down all ER sensors and eIF2a, an essential ER stress transducer, using double strand RNAs (dsRNAs) and measured TOR activity. Each dsRNA was transfected into the S2R+ cells and incubated for two days; then, the cells were collected without ER stress induction, and P-S6K level was measured by Western blot. DsRNA of luciferase (Luc) was used as a baseline. DsRNA of TSC1, a TOR pathway upstream inhibitor, served as a positive control. TSC1 dsRNA increased the level of P-S6K when compared to that in the cells transfected with control dsRNA (Luc) (Figure 3). However, knock down of eIF2a, a crucial PERK target, did not affect S6K phosphorylation. Among the three ER sensors, Atf6 dsRNA most significantly reduced the level of P-S6K. PERK dsRNA also reduced the level of P-S6K, but to a lesser extent. We further investigated how Atf6 regulated the TOR pathway since knocking down of Atf6 reduced P-S6K the most (Figure 3).

### Overexpression of Atf6 activates the TOR pathway

**Figure 4:**
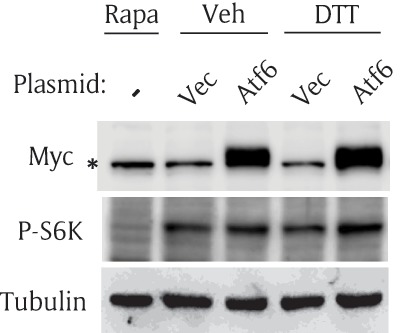
**Overexpression of Atf6 activates the TOR pathway.** S2R+ cells were transfected with an empty vector (Vec) or myc-Atf6 construct (Atf6); then, cultured for 2 days. The cells were incubated with vehicle or DTT in serum-free media for one hour. The cells were collected and subjected to Western blot. Rapamycin (Rapa) serves as a negative control for P-S6K. Asterisk (*) indicates a non-specific band in myc blot.

Since knock down of Atf6 inhibited the TOR pathway (Figure 3), we further tested whether overexpression of Atf6 can activate the TOR pathway. We generated a plasmid (pAc5.1-myc-Atf6) to express myc-Atf6 fusion protein under the control of the *Drosophila* actin promoter. S2R+ cells were transfected with either an empty vector or a myc-Atf6 vector and further cultured for two days. Myc-tagged Atf6 fusion protein was expressed at an expected size of 100 kD, and myc-Atf6 increased the phosphorylation of S6K regardless of DTT treatment (Figure 4). To enhance the effect of Atf6 overexpression, we did not pre-incubate the transfected cells in nutrient-free media (NF-M) before DTT treatment. Thus, phosphorylation of S6K was not reduced to basal levels. Of note, we found that DTT minimally affected P-S6K in this experimental condition. The levels of P-S6K in the vector were comparable in both the vehicle (lane 2) and in DTT (lane 4).

### Atf6 is necessary for ER stress-mediated TOR activation

**Figure 5:**
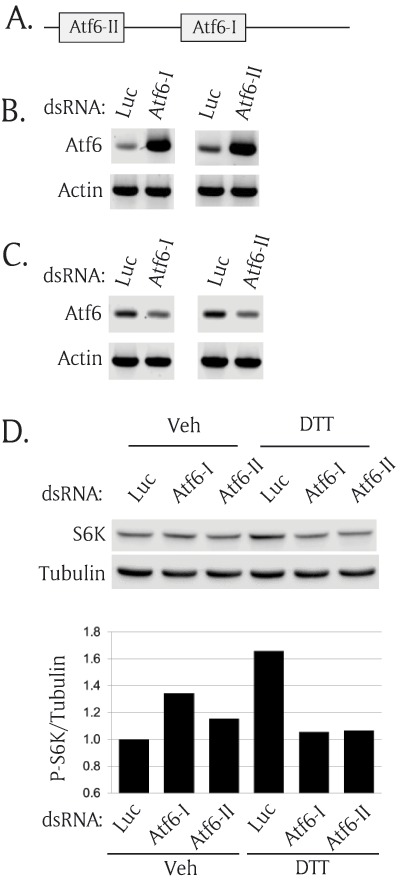
**Atf6 is necessary for ER stress-mediated TOR activation. (A)** A schematic diagram of two regions of Atf6 encoding double strand RNA (dsRNA) **(B)** Transfected Atf6 dsRNAs were present inside the cells 2 days after transfection. S2R+ cells were transfected with two regions of Atf6 dsRNAs and cultured for 2 days. The total RNAs were extracted and used to generate cDNA. Reverse transcriptase (RT)-PCR was performed to detect the transfected dsRNA by amplifying the inside sequence of the transfected dsRNA. Actin serves as endogenous control. **(C)** Atf6 dsRNAs knocked down endogenous Atf6 expression. To detect only Atf6 mRNA, the outside region of the transfected dsRNA was amplified using RT-PCR. Actin serves as an endogenous loading control. **(D)** Knock down of Atf6 abolished ER stress-induced phosphorylation of S6K. S2R+ cells were transfected with two different regions of Atf6 dsRNA. The transfected cells were cultured for 2 days and incubated with nutrient-free media for 1 hour. Then, the cells were incubated with vehicle or 1 mM DTT. The cells were collected and subjected to Western blot analysis. The Western blot results were quantitated and shown as the ratio of P-S6K/tubulin.

Since Atf6 is necessary to activate the TOR pathway (Figure 3), we hypothesized that knocking down of Atf6 would abolish ER stress-mediated activation of the TOR pathway. To test the hypothesis, we transfected S2R+ cells with Atf6 dsRNA, induced ER stress, and measured the levels of P-S6K in the transfected cells. To rule out off-target effects of dsRNAs, we designed another Atf6 dsRNA to target a different region of Atf6 (denoted by “Atf6-II”). We named the original Atf6 dsRNA “Atf6-I” as shown in Figure 5A. First, we tested whether the dsRNA was present in the transfected cells two days after transfection using reverse transcription (RT)-PCR. Using primer sets amplifying Atf6 mRNA and dsRNA, we found that both Atf6 dsRNAs (Atf6-I and II) were present inside the transfected cells for at least two days after transfection (Figure 5B). We then demonstrated both dsRNAs successfully knocked down Atf6 mRNA using RT-PCR with Atf6 mRNA-specific primer sets (Figure 5C). As hypothesized, knocking down of Atf6 completely eliminated ER stress-mediated activation of P-S6K (Figure 5D). The cells transfected with control dsRNA (Luc) showed DTT-induced S6K activation, but Atf6 dsRNA-transfected cells completely abolished S6K activation. To quantify the Western blot, we used Odyssey FC imager and measured band intensities of P-S6K and tubulin. Each bar graph represents P-S6K normalized by corresponding tubulin in the Western blot (Figure 5D). This data further demonstrate that Atf6 is a critical mediator of the ER stress-TOR signaling axis.

### Both S1P and S2P are necessary to activate ER stress-mediated activation of the TOR pathway

**Figure 6:**
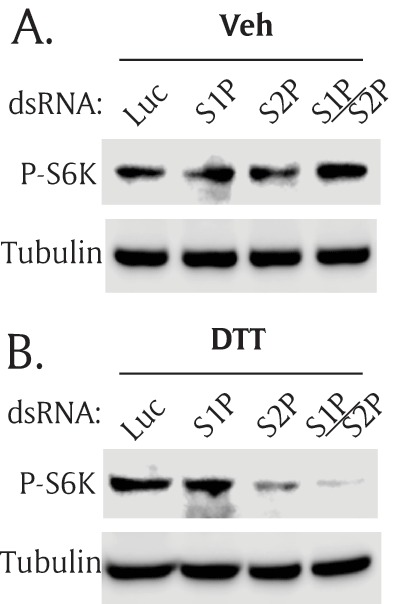
**Both S1P and S2P proteases are necessary for ER stress-mediated TOR activation.** S2R+ cells were transfected with double-strand (ds) RNA of S1P and S2P, separately or together. The transfected cells were cultured for 2 days, and treated with serum-free media **(A)** without ER stress, or **(B)** with ER stress. Then, Western blot analysis was performed with phospho(Thr398)-specific S6K antibody or tubulin antibody. Site-1 protease (S1P), Site-2 protease (S2P).

In response to ER stress, Atf6 is translocated from ER membrane to the Golgi apparatus where it is processed by two Golgi membrane proteases, site-1 protease (S1P) and site-2 protease (S2P) [11]. The cleaved Atf6 transcription domain is released to the cytoplasm, moves into the nucleus, and regulates its target genes to relieve ER stress. Since S1P and S2P are necessary to activate Atf6 during ER stress, we hypothesized that S1P and S2P would be necessary for ER stress-mediated activation of the TOR pathway. S1P and S2P dsRNAs were generated and transfected separately or together into S2R+ cells. Without ER stress, knocking down of S1P and S2P did not affect P-S6K (Figure 6A). However, when ER stress was induced, knocking down of both S1P and S2P significantly inhibited phosphorylation of S6K (Figure 6B). These data suggest that S1P and S2P affect the TOR pathway largely via Atf6 not via other S1P/S2P substrates [11,32]. Of note, knocking down of either S1P or S2P alone did not consistently affect P-S6K although S2P silencing seemed more effective. These data suggest that transcriptional activity of Atf6 is necessary to achieve the ER stress-mediated activation of the TOR pathway.

## Discussion

The ER stress response pathway and the TOR pathway regulate functionally equivalent cellular processes such as protein synthesis, lipid metabolism, and cell survival [3,19]. Interconnection between the two signaling pathways has been explored and described [22], but the detailed mechanism is far from clear. To test the possible regulation of TOR signaling by ER stress, we treated S2R+ *Drosophila* cells with ER stress-inducing drugs and then, assessed their TOR activities. Although the three drugs induce ER stress through different mechanisms, they all activated the TOR signaling pathway (Figure 2). This result indicates that ER stress may function as an upstream activator of TOR signaling cascade. However, the ER stress pathway could inhibit the TOR signaling pathway depending on cellular contexts such as different cell types and duration of ER-stress inducing drug treatment [23]. This paradox might be explained through Akt (also known as PKB), a positive regulator of the TOR pathway since Akt activity oscillates during the course of ER stress [33]. Upon ER stress, Akt is instantly activated but slowly inactivated as the stress progresses. Thus, it is plausible that the TOR activation might be synchronized with Akt activity. Inversely, TOR signaling controls the ER stress pathway. For example, uncontrolled activation of the TOR pathway initiates ER stress through overwhelming protein synthesis [34]. Conversely, inhibition of TOR signaling prevents palmitate-induced ER-stress in 3T3-L1 adipocytes and blocks ER stress-induced apoptosis in HepG2 carcinoma cells [26,35].

With loss of function and gain of function analyses, we identified that Atf6 is a major stress-responsive ER sensor, which connects ER stress and the TOR signaling pathway (Figures 3 and 4). Atf6 is an ER membrane-bound transcription factor, and it is activated by proteolysis to produce an active transcription factor in response to ER stress [11,36]. The activated Atf6 transcriptionally controls various genes functioning in protein folding and the maintenance of ER homeostasis [12,37]. In addition, Atf6 regulates numerous genes in lipid metabolism; specifically, it stimulates cholesterogenic and lipogenic genes by antagonizing sterol regulatory element-binding protein 2 (SREBP2) [38,39]. Supporting this notion, Atf6 null mice are more insulin-sensitive and contain less serum triglyceride (TG) [40]. These data are reminiscent of renal transplanted patients treated with rapamycin, a potent TOR inhibitor. The TOR inhibitor increases serum TG levels in the patients [41]. Further, S6K null mice are insulin-sensitive and resist diet-induced obesity [29]. These virtually mirroring phenotypes highlight Atf6’s role as a molecular linker between the two signaling pathways. We demonstrated that ER stress inducers failed to activate the TOR pathway when expression of Atf6 was inhibited (Figure 5D).

Our data support the notion that ER stress activates TOR signaling and inversely, activated TOR pathway can induce ER stress [34]. Then, what would be the physiological role of this positive feedback loop in the ER stress-TOR pathway axis? The primary response of the UPR is to remove accumulated unfolded/ misfolded proteins and reduce new ER load through proteolytic protein degradation and inhibition of protein synthesis. However, in the second stage, the stressed cells need to overcome protein synthesis block and maximize protein synthesis in order to recover from the stress. The TOR pathway serves as a central regulator of protein translation [42]. Two main targets of TOR, 4EBP and S6K, stimulate protein translation by activating translation initiation of eIF4E and upregulating ribosome biogenesis, respectively [42,43]. Thus, this positive feedback loop between ER stress and the TOR pathway could maximize their protein synthesis and recover from the primary stress response, protein synthesis block.

We have shown that S1P and S2P proteases are essential to induce activation of the TOR pathway during ER stress (Figure 6). Nakajima et al. has shown that an inhibitor of S1P and S2P proteases, 4-(2-aminoethyl) benzenesulfonyl fluoride (AEBSF) inhibits the TOR pathway [44]. Thus, our and other group’s data evidently demonstrate that Atf6 transcriptional activity is necessary for ER stress-mediated TOR signaling activation. Then, what are the Atf6 target molecule(s) responsible for activating TOR signaling? Among the identified target molecules, Rheb and Notch 1 could be good candidates to connect the two pathways since both Rheb and Notch1 can activate the TOR pathway [45,46]. Another possibility is that Atf6 might indirectly activate the TOR pathway through the Akt/TSC/TOR axis [47], since Atf6 is shown to activate Akt [44]. However, crosstalk between the two signaling pathways may not be simply linear. Instead, the two pathways are intertwined with other signaling pathways such as autophagy and the cell death pathway requiring further investigation for complete understanding of crosstalk between the two signaling pathways.

## Abbreviations

Activating transcription factor 6 (Atf6), DEP-domain-containing TOR-interacting protein (Deptor),eukaryotic initiation factor 2a (eIF2a), Endoplasmic reticulum (ER), ER-associated degradation (ERAD), eukaryotic initiation factor 4E binding protein 1 (4E-BP1), Inositol requiring enzyme 1 (IRE1), Mammalian lethal with Sec13 protein 8 (mLST8), Pancreatic ER kinase (PERK), Proline-rich AKT substrate 40 kD (PRAS40), Ras homolog enriched in brain (Rheb), Regulated IRE1-dependent mRNA decay (RIDD), Regulatory-associated protein of TOR (Raptor), Ribosomal protein S6 kinase (S6K), Site-1/2 proteases (S1P/S2P), Target of rapamycin (TOR), Tuberous sclerosis complex 1/2 (TSC1/2), Unfolded protein response (UPR), X-box binding protein-1 (XBP1).
